# *ShelXle*: a Qt graphical user interface for *SHELXL*

**DOI:** 10.1107/S0021889811043202

**Published:** 2011-11-12

**Authors:** Christian B. Hübschle, George M. Sheldrick, Birger Dittrich

**Affiliations:** a Institut für Anorganische Chemie, Georg-August-Universität Göttingen, Göttingen, Germany

**Keywords:** molecule viewers, electron density maps, syntax highlighting, isosurfaces, *SHELX*, *SHELXL*, graphical user interfaces

## Abstract

*ShelXle* is a graphical user interface for *SHELXL* [Sheldrick, G. M. (2008). *Acta Cryst.* A**64**, 112–122], currently the most widely used program for small-molecule structure refinement. It combines an editor with syntax highlighting for the *SHELXL*-associated .ins (input) and .res (output) files with an interactive graphical display for visualization of a three-dimensional structure including the electron density (*F*_o_) and difference density (*F*_o_–*F*_c_) maps. Special features of *ShelXle* include intuitive atom (re-)naming, a strongly coupled editor, structure visualization in various mono and stereo modes, and a novel way of displaying disorder extending over special positions. *ShelXle* is completely compatible with all features of *SHELXL* and is written entirely in C++ using the Qt4 and FFTW libraries. It is available at no cost for Windows, Linux and Mac-OS X and as source code.

## Abstract 2

*ShelXle* is a user-friendly graphical user interface for *SHELXL*. It combines an editor with syntax highlighting for *SHELXL*-associated files with an interactive graphical display for visualization of a three-dimensional structure.

## 1. Introduction

The *SHELX* programs as originally developed in the 1970s were intended for use with photographic intensity data, punched cards and computers multiple orders of mangitude slower than even the most basic models on the market today (Sheldrick, 2008 ▶). In the early days of *SHELX*, a crystal structure refinement usually involved examining a lineprinter output – *i.e.* drawing lines between the numbers to create a ‘picture’ of the structure – followed by editing a few of the input and output cards with a card-punch and combining the cards to create the input deck for the next refinement job, which usually ran overnight. The way crystal structure determinations are performed today is clearly different, but – somewhat surprisingly – *SHELXL* is still used in most small-molecule structure refinements.

More recently, a number of excellent graphical user interfaces (GUIs) [*e.g. WINGX* (Farrugia, 1999 ▶), *OLEX2* (Dolomanov *et al.*, 2009 ▶), *XSEED* (Barbour, 2001 ▶), *PLATON* and *SYSTEM-S* (Spek, 2009 ▶), and the Bruker programs *XP* (Nicolet, 1981 ▶) and *XSHELL* (Bruker, 2000 ▶)] have been developed to facilitate structure refinement with *SHELXL* as the underlying program, but in general the punched-card way of thinking that was central to the design of *SHELXL* has proven awkward to integrate into a modern interactive computer-graphics environment without losing at least some of the unique capabilities of the original program. Despite the availability of a very informative International Union of Crystallography monograph (Müller *et al.*, 2006 ▶) describing the application of *SHELXL*, we felt that there was still a need for a simple, intuitive and robust GUI that uses state-of-the-art programming techniques but retains as much as possible of the original *SHELX* flavour and capabilities. For this purpose, *ShelXle* was developed. *ShelXle* shares some concepts with earlier programs, such as *MoleCoolQt* (Hübschle & Dittrich, 2011 ▶), but most of the code was rewritten.

## 2. Technical description and functionality

**Figure 1:**
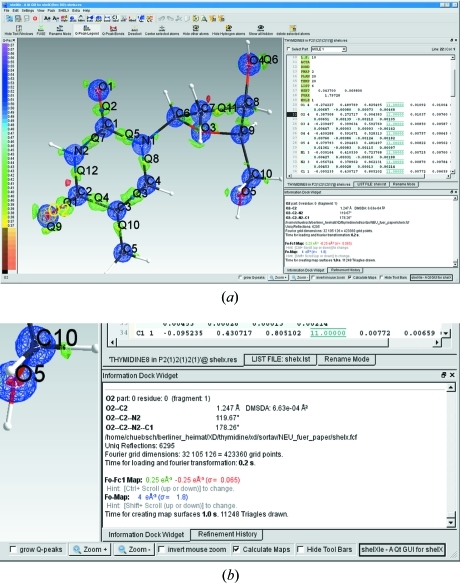
(*a*) The GUI of *ShelXle*, displaying the structure of thymidine at 20 K (Hübschle *et al.*, 2008 ▶). The *F*_o_–*F*_c_ map at 0.25 e Å^−3^ shows features of bonding and lone-pair electron density. Atom O2 is currently selected. (*b*) An enlargement of the lower-right corner of the screenshot above.

*ShelXle* opens a *SHELX*-format .res file from a structure solution program or a *SHELXL* refinement. The .ins/.res file in *SHELX* format is shown in an interactive editor window (on the right side of the graphical interface) and (on the left side) the mono or stereo visualization of the three-dimensional structure is displayed. The display and editor are strongly coupled. The editor uses colour highlighting to identify the currently chosen atom and also possible syntax errors. Clicking on an atom in the displayed structure moves the text cursor to the corresponding atom in the editor. An atom can also be selected by right clicking on a line in the editor containing an atom, which is then centred in the display. The GUI is controlled by menus and toolbars; command-line input is neither required nor implemented. Fig. 1 ▶ gives a general impression of the appearance and functionality of *ShelXle. ShelXle* is written entirely in C++ using the Qt4 (http://qt.nokia.com/products/) and the FFTW (http://www.fftw.org/) libraries, and so is able to exploit the latest developments in computer graphics as well as being highly portable.

### 2.1. Electron density maps

If the previous *SHELXL* refinement used the ‘LIST 6’ instruction, *F*_o_ and *F*_o_–*F*_c_ maps are calculated and visualized as mesh-style isosurfaces. The colour scheme used is the same as in the program *COOT* (Emsley *et al.*, 2010 ▶). The isocontour level of such maps can be controlled by using either the mouse wheel or a dialogue window. The contour level of the difference map may be changed with the mouse wheel while pressing the control key (or the command key under Mac-OS), and the contour level of the *F*_o_ map is changed in the same way but using the shift key. Initial isocontour levels are 2.7σ for the *F*_o_–*F*_c_ map and 1.2σ for the *F*_o_ map, where σ is the square root of the average variance of the density. These maps are in principle infinite in all directions, but the region displayed is restricted by clipping planes perpendicular to the viewing direction. If deemed desirable, in order to simplify the view, it is possible to display only density within 1.41 Å (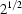) of any visible atom or ‘*Q* peak’ (difference electron density peak from *SHELXL*).

It may sometimes occur that the parameters of the *SHELXL* PLAN instruction are not sufficient to generate a *Q* peak at a desired position, for example when dominant heavy atoms are present. In such cases *ShelXle* can generate further *Q* peaks by searching for peaks in the *F*_o_–*F*_c_ residual density that are higher than the current isosurface value.

### 2.2. Special handling of difference electron density maxima (‘*Q* peaks’)

*Q* peaks are visualized as small colour-coded icosahedra. The colour of a *Q* peak corresponds to the peak height. A separate *Q*-peak list shows the correspondence between colours and peak heights. By moving the mouse over this list, labels of *Q* peaks with the same peak height are highlighted. If the mouse pointer hovers over a *Q* peak, the region representing its height is highlighted in the list. *Q* peaks below a certain threshold may be hidden temporarily by clicking on the *Q*-peak list. Once some of the *Q* peaks have been hidden in this way, the cutoff value can be adjusted by scrolling with the mouse wheel while the mouse pointer is over the list.

### 2.3. Adding H atoms

**Figure 2:**
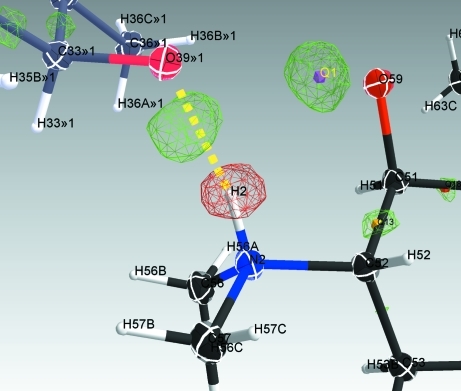
An early refinement state of roxithromycin (Holstein *et al.*, 2010 ▶), showing difference electron density. Missing and erroneously placed H atoms are clearly visible.

The ‘add H atoms’ function in *ShelXle* places hydrogen atoms automatically by generating the corresponding AFIX instructions in the file being edited. If the *F*_o_–*F*_c_ map is available, the difference electron density may be used to find optimal positions for H atoms in CH_3_ groups in a similar manner to the way in which the ‘HFIX 137’ command in *SHELXL* operates. As methyl groups are often disordered, there is a facility to place six H atoms in idealized positions and to refine an occupancy parameter to describe the disorder using one additional free variable that is generated automatically. Fig. 2 ▶ illustrates the usefulness of the difference electron density in placing the H atoms correctly.

### 2.4. The editor: syntax highlighting and codeword completion

One of the core functionalities of *ShelXle* is the editor and its ability to perform syntax highlighting. All known *SHELXL* commands are highlighted in the same way (dark red over light green). Permanent comments (REM cards or following ‘!’) are coloured in blue, while temporary comments (lines beginning with a space when the line before does not end with ‘ = ’) are coloured dark blue and are underlined. Lines longer than 80 characters are flagged by a red background colour, since characters after column 80 (not compatible with punched cards) are ignored by *SHELXL*. After the first one or two characters of a new line have been entered, a code-completer function opens, suggesting commands beginning with the given letters. Accepting a suggestion by striking the ‘enter’ key inserts the command in capital letters (whether or not they were entered in upper case).

Care is taken to keep track of the ‘free variables’, a defining feature of *SHELXL*. When a number in the editor window implicitly references a free variable and the mouse pointer hovers over it for several seconds, a popup window appears with the interpretation.

Analogously, a brief description of each *SHELXL* command is given when the mouse is placed over a line starting with a command. If lines containing atoms are selected in the editor, right clicking in the selected area in the editor achieves atom selection. The editor is also equipped with a ‘search and replace’ tool that highlights matches in the editor in yellow. Entire parts (‘PART’) and residues (‘RESI’) can also be selected. This function allows the selection of disordered PARTs, either separately or in combination with the ordered PART. Unselected atoms can optionally be hidden. A residue may also be selected using a residue list. In addition, facilities are provided for rearranging the windows. When desired, or prior to performing a refinement, the three-dimensional display and the editor are synchronized and the editor contents are saved. More esoteric *SHELXL* instructions – *e.g.* FRAG…FEND or the third number on the L.S. command – can easily be added using the editor.

### 2.5. Refinement history facility

Like *OLEX2* (Dolomanov *et al.*, 2009 ▶), *ShelXle* is equipped with a refinement history, where every refinement step is saved and represented within the GUI by a bar. The colour and height of the bar symbolize the *R* value of each refinement step. By left clicking on a bar, a particular refinement step can be loaded into the editor and displayed graphically. In this way users can go back to a previous refinement step, which can be useful if the refinement becomes unstable or the strategy employed proved to be a dead end. The refinement history can be pruned or a preview can be displayed by right clicking. In addition to the refinement history, *ShelXle* stores a backup each time the editor contents are saved. One of these backup versions can be selected in a dialogue window; this dialogue also contains a preview, where every line that is different from the current version of the file is highlighted in dark orange. All history files are stored in a subdirectory called ‘shelXlesaves’, which is placed in the directory where the structure file is located. *ShelXle* does not generate hidden or write-protected files or directories.

### 2.6. The information window

All text output is collected and displayed in the information window. Hydrogen bonds found in the structure are tabulated. The contents of this window are stored internally as HTML; any part of it can easily be marked, copied and pasted to word-processing programs. Distances, angles, torsion angles and the differences of mean-square displacement amplitudes (DMSDAs; Hirshfeld, 1976 ▶; Rosenfield *et al.*, 1978 ▶) may be displayed in the information window. Where appropriate, crystallographic symmetry operators are displayed. Values of free variables and how often they are used can also be found in the information window, as can statistical details of the electron density maps.

### 2.7. Labelling and renaming atoms

When ‘rename mode’ is selected, a popup window displays the element type, a numerical index and a suffix. By clicking on an atom or a *Q* peak, these values are applied to that atom and the numerical index is increased by one. It is also possible to use this mode to set PART numbers, residue numbers and residue names. When PART numbers not equal to zero are applied, there is an option to tie the occupancy to a free variable (or to one minus the free variable). If the free variable is not yet defined, it is generated and inserted into the FVAR instruction. The original atom record is retained as a temporary comment. If an existing atom has the same combination of atom name, PART and RESI number, the colour of the new atom name changes to red to warn the user; when the combination is unique the atom name is green. The user is allowed to create duplicate atoms but should resolve such *SHELXL* incompatibilities before starting the next refinement.

When there is more than one chemically identical molecule in the asymmetric unit, *ShelXle* provides an option to label subsequent molecules in the same way as the first. Each identical molecule is assigned a different residue number. This is achieved in a semi-automated manner in which the user can assign atoms of the target molecule to be equivalent to specified atoms of the source molecule 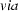 a ‘drag and drop’ dialogue.

**Figure 3:**
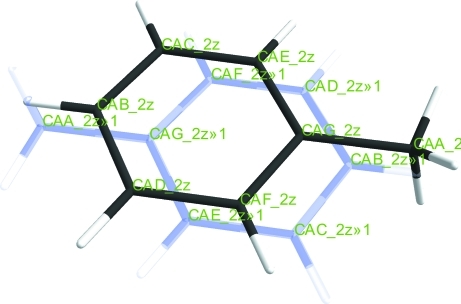
A toluene molecule disordered over an inversion centre refined in a ‘PART -1’ environment. The symmetry-equivalent molecule is visualized as a pale-blue ‘ghost’ molecule.

Fig. 3 ▶ illustrates the way in which a disordered ‘PART -1’ molecule lying close to a symmetry element is displayed.

### 2.8. Other convenient functions

The built-in editor gives the user full control over the *SHELXL* input file. This means that more advanced *SHELXL* commands can be added directly. Nevertheless, many tasks in routine structure refinement can be repetitive and time consuming, so *ShelXle* provides the functionality to expedite some of them. Examples are applying the suggested weighting scheme or updating the cell contents in the UNIT instruction to make it consistent with the atom list.

After all necessary changes have been made, selecting the appropriate menu option or pressing the function key ‘F2’ causes the currently edited .res file to be saved as a .ins file and a *SHELXL* refinement job to be started. The refinement can be followed in an output window, with important items highlighted to improve readability. On completion of the refinement the user can choose between updating the editor window or discarding the results; updating is blocked to prevent accidents if a critical error has occurred in the refinement.

Sometimes it happens that molecules lie outside the primary unit cell with 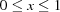, 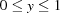, 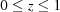. Often this is first noticed on checking the refinement results with *checkCIF* (http://checkcif.iucr.org). Optionally *ShelXle* applies an algorithm to move the centre of gravity of each molecule into the primary unit cell, whilst ensuring that the molecules are as close as possible to each other. It is also possible to call additional programs from within *ShelXle*, *e.g*. *PLATON* (Spek, 2009 ▶) to perform diagnostics before the refinement is completed or to make *ORTEP* (Johnson & Burnett, 1996 ▶) style plots *etc.* as an alternative to the screenshot provided by *ShelXle*.

## 3. Program availability

*ShelXle* is available at no cost for most modern Windows, Linux and Mac-OS X systems. For details on how to obtain *ShelXle*, see http://www.moliso.de/shelxle/.
